# Inter-species stimulus enhancement: herring gulls (*Larus argentatus*) mimic human food choice during foraging

**DOI:** 10.1098/rsbl.2023.0035

**Published:** 2023-05-24

**Authors:** Franziska Hacker, Kiera Smith, Paul Graham

**Affiliations:** School of Life Sciences, University of Sussex, Brighton, East Sussex, BN1 9QG, UK

**Keywords:** social learning, stimulus enhancement, herring gull, kleptoparasitism, inter-species learning, human–wildlife interaction

## Abstract

Herring gulls (*Larus argentatus*) are one of few species that thrive in anthropogenic landscapes, and their familiarity with people makes them an excellent target for studies of inter-species social cognition. Urban gulls pay attention to human behaviour in food-related contexts and, thus, we set out to investigate whether such cues can influence a gull's attention to and choice of potential food items in their environment. Herring gulls were given free choice of two differently coloured anthropogenic food items in the presence of a demonstrator, who was either sitting still or eating food from an item that matched one of the presented ones. We found that a demonstrator eating significantly increased the likelihood of a gull pecking one of the presented items. Furthermore, 95% of pecks were directed toward the presented food item that colour-matched the demonstrator's food item. The results showed gulls were able to use human cues for stimulus enhancement and foraging decisions. Given the relatively recent history of urbanization in herring gulls, this cross-species social information transfer could be a by-product of the cognitive flexibility inherent in kleptoparasitic species.

## 1. Introduction

Some species, such as the European herring gull (*Larus argentatus*), thrive in anthropogenic environments and, following the first instances of urban nesting in the 1940s, urban colonies have spread across the UK [1] despite declining overall population numbers [2]. Herring gulls are generalist predators and kleptoparasites that will feed on intertidal marine organisms [3] and steal prey from both hetero- and conspecific individuals [4–8]. Additionally, urban herring gulls are familiar with the presence of people and the foraging opportunities they provide [9,10]. This success in urban environments is suggested to result from behavioural flexibility [7,11–14], which is likely to require specific cognitive adaptations. In food-stealing birds, success is said to reflect an ability to integrate and use information about both the environment and other individuals [15], and kleptoparasites generally have larger relative brain sizes than their hosts [15].

Herring gulls are social foragers and their long lifespan and 4-year immature period [16] could provide ample opportunities for extensive social learning, which is common among animals with a long developmental stage [17–20]. Gulls frequently obtain food after watching others flock to a food source [21], suggesting they make use of social learning to identify the relationship between a stimulus and a demonstrator, shaping future behaviour [22]. Interestingly, in urban populations there is evidence that herring gulls can also obtain foraging information from humans. Recent studies have found that gulls adapt their foraging behaviour to human activity patterns [23], increase their attention towards a person in possession of food [24], and pay attention to behavioural cues, such as gaze [8,25] or whether items have been handled [26]. This shows that gulls have the cognitive and behavioural flexibility to adapt their foraging behaviour to human cues. However, previous studies have focussed on whether gulls identify and show interest in people in possession of food, or on their response to directly handled and presented food items, but not their broader observational skills and cognition beyond the ability to track objects.

We further studied the cognitive abilities of gulls by asking if they can pay attention to humans and anthropogenic food items and transfer that knowledge to a food choice via social observation. Using a stimulus enhancement assay, in which a demonstrator's activity allows an observer to learn about a particular object class [27], we asked if the details, herein the colour, of a food item being consumed by a human can influence a herring gull's choice between two presented food items.

## 2. Methods and materials

### (a) Study site

Data were collected during daylight hours (07.00–16.00) along the 8.7 km long Brighton beachfront, UK (50.8193°N, 0.1364°W), from May–June 2021 (F.F.) and March–May 2022 (K.S.). Relying on this species site fidelity [10,28,29], experimental locations were shifted between trials and days to minimize the chance of retesting individuals, with no surveys being conducted at the exact same location and that of subsequent trials being spaced at least 200 m apart. Low tide periods were avoided as pilot studies showed that gulls would be more preoccupied with natural foraging and thus less likely to engage. Furthermore, data were only collected on weekdays to minimize pedestrian disturbance.

### (b) Data collection

**Figure 1.**
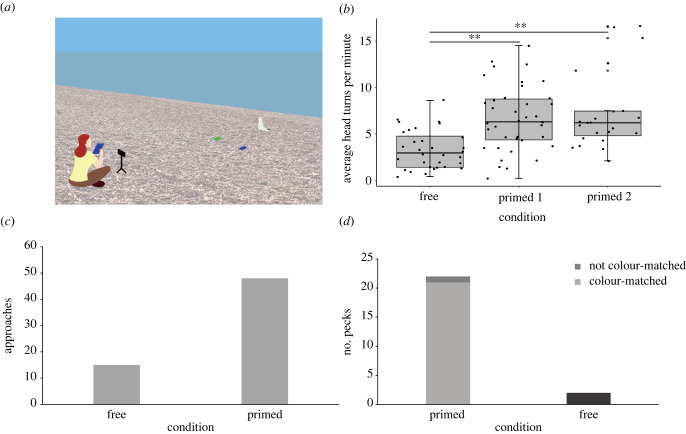
Human behaviour biased gull attention and foraging behaviour. (*a*) Illustration of the experimental set-up. After placing the crisp packets (blue and green squares) in the vicinity of the target gull, the experimenter retreated behind the camera and either remained like that (FC) or retrieved their own packet of blue or green crisps (PC1 and PC2, respectively; later PC). (*b*) The average number of head turns per minute in FC (*n* = 32), PC1 (*n* = 38), and PC2 (*n* = 23) trials (2022 data only). Black dots show actual data points, with box plots highlighting the medians and 1^st^ and 3^rd^ quartiles. *Post-hoc* Mann–Whitney *U* comparisons with significance at *p* < 0.001 are marked as ** (FC versus PC1: W = 252, *p* < 0.001; FC versus PC2: W = 125, *p* < 0.001; PC1 versus PC2: W = 429.5, *p* = 0.917). (*c*) Approaches observed in PC (*n* = 103) and FC (*n* = 80) trials (2021 and 2022 data). (*d*) Pecks in the PC (22 pecks, *n* = 103) and FC (two pecks, *n* = 80) conditions (2021 and 2022 data). Pecks in the PC condition were further categorized by whether they colour-matched the primary food item held by the human experimenter.

Previous work [24] has shown that group size does not influence the frequency of attentional markers; thus, single individual herring gulls and groups of less than five were approached after assessing the area to ensure there were no dogs or pedestrians moving in the immediate vicinity. The presented stimuli were two Walkers brand crisp packets, one blue, one green, taped to 15 cm × 20 cm ceramic tiles, which were laid out 1.5 m apart and 5–10 m away from the gulls. The left–right position of the packets was alternated to control for side bias. The experimenter retreated and sat on the ground approximately 5 m from the crisp packets. Subjects were recorded on an iPhone XS or Honour 20 mounted to a LINKCOOL tripod (figure 1*a*). Recording was stopped when a gull pecked at one of the food items or after 5 min had passed, unless a gull was approaching at the 5 min point. No individuals were forced to engage with the experimenter and all gulls had the freedom to remove themselves at any time. We adhered to the Association for the Study of Animal Behaviour (ASAB) guidelines [30], and all necessary ethical approval was obtained from the University of Sussex.

We recorded the gulls' behavioural responses to the experimenter and presented stimuli in three conditions. The free condition (FC) involved the experimenter retreating and sitting neutrally, holding a fixed gaze directed at the camera screen to control for human gaze cues [8]. Primed condition 1 (PC1) involved the experimenter retrieving a blue crisp packet from their bag and eating from it, while primed condition 2 (PC2) involved the experimenter retrieving and eating from a green crisp packet. The trial order was predetermined.

### (c) Video analysis

Recordings were uploaded into the Behavioural Observation Research Interactive Software (BORIS) [31] and a log of head turn counts was created, while approaches and pecks were noted as ‘yes’ or ‘no’ along with whether they colour-matched the experimenter's item during PC trials. These behaviours were chosen based on previous work showing that they are upregulated when a demonstrator is consuming food rather than simply mimicking feeding actions [24]. For an individual to be included in the analysis it had to be present in the video for 30 s or more or peck at one of the provided crisp packets. When more than one gull approached, only the first subject was included in the analyses. A head turn was recorded if the gull turned its head toward the experimenter, and the average number of head turns per minute per individual was calculated; head turn data were only obtained in 2022, resulting in *n* = 93 for all head turn analyses. Approaches were defined as the gull moving from its original position to at least halfway towards the food items, and a peck was recorded if the gull's beak came into contact with a crisp packet. Approach and peck data were collected in 2021 and 2022, resulting in *n* = 183. Data were exported from BORIS into Microsoft Excel and statistical tests (Shapiro–Wilk, Kruskal–Wallis, Mann–Whitney *U*, Pearson's chi-squared and Fisher's exact tests) as well as visualizations were performed in R v. 4.0.3 [32].

Lastly, the frequency of attentional markers [24] was compared between adults and immature individuals. The age of the gulls tested in both years was recorded as immature or adult based on their plumage; any individual with brown markings, irrespective of any grey wing feathers, was recorded as immature. The approach directness of a subset of the approach data from both years (58 out of 64 approaches) was ranked from 1–5 by an independent observer, naive to the experimental condition, with 1 being the least and 5 being the most direct.

## 3. Results

### (a) Human behaviour impacts gull attention

We first aimed to establish the impact of human food cues on three markers of attention: head turns, approaches and pecks. For this, we first investigated the potential colour-bias by comparing the responses in FC, PC1, and PC2 trials. All three attentional markers were increased during PC1 and PC2 compared to FC but did not differ significantly between PC1 and PC2 (electronic supplementary material, table S1; figure 1*c* shows head turns as an example); thus, we combined data from the two primed conditions (PC) for further analysis.

Overall, head turns were more frequent in PC versus FC trials (Kruskal–Wallis chi-squared = 23.476, d.f. = 1, *p* < 0.001) and, similarly, approaches (figure 1*c*) occurred significantly more often in PC trials (Pearson's chi-squared test, *x*^2^ = 16.448, d.f. = 1, *p* < 0.001), with 47.57% of birds approaching during PC compared to 18.75% during FC trials. Furthermore, pecks (figure 1*d*) happened significantly more often in PC compared to FC trials (Fisher's exact test, *p* < 0.001), with 21.36% and 2.50% of birds pecking at the crisp bags, respectively.

We further analysed markers of attention by age (electronic supplementary material, figure S1). The average number of head turns did not differ significantly between adult and immature birds. In FC trials, we recorded a median of 3.35 head turns per minute for adults and 2.75 for immature individuals (Mann–Whitney *U* test, *W* = 113.5, *p* = 0.460); in PC trials the medians were 6.45 and 5.90, respectively (Mann–Whitney *U* test, *W* = 525.5, *p* = 0.271). Similarly, the number of approaches (Pearson's chi-squared test, *x*^2^ = 2.627, d.f. = 1, *p* = 0.105) did not differ significantly between age groups, irrespective of condition. By contrast, the likelihood of pecking did differ significantly with age during PC trials (Fisher's exact test, *p* < 0.001), with 86.36% of pecks coming from adults, which made up only 46.60% of the birds observed in PC trials.

### (b) Human food cues influence gull foraging choices

We further asked if gulls observe human food choice and use that information to redirect their attention toward a specific stimulus and mimic human food choice. We observed that approaches that resulted in a peck (37.50%) were very strongly biased to the packet that colour-matched the food item held by the experimenter (95.45% of pecks), which was confirmed by a Pearson's chi-squared test investigating the correlation between the gull's choice (blue/green bag) and the colour of the hand-held item (blue/green bag) (*x*^2^ = 18.333, d.f. = 1, *n* = 22 pecks, *p* < 0.001). Furthermore, the directness analysis on a subset of approaches showed that gulls approached a presented food item (crisp packet) more directly in PC trials compared to FC trials (Mann–Whitney *U* test, *W* = 57, *n* = 58, *p* < 0.001).

While our above presented results clearly indicate that gulls will show interest in an anthropogenic food item on the ground, at other times the same objects are ignored. During FC trials (*n* = 80), a total of 15 gulls approached (18.75%), but only two (2.50% of total or 13.33% of approaching birds) pecked at the crisp bags on the ground. In comparison, during PC trials (*n* = 103), 49 gulls approached (47.57%) and 22 (21.36% of total or 44.90% of approaching birds) pecked at one of the food items. Ten (9.71% of total or 20.41% of the approaching birds) ignored the crisp bags on the floor and walked past them, approaching the experimenter instead, which never happened in FC trials.

## 4. Discussion

While comparative attempts to categorise or rank the cognition of gulls relative to other animals have been inconclusive [33–36], it is clear from naturalistic foraging tasks that gulls have rich behavioural repertoires, suggestive of a high level of cognition. Observations of herring gulls dropping shells demonstrate persistence, concentration, and mental representations of the distribution of drop sites [33]; systematic food handling reflects skill and purposefulness [33]. Furthermore, urban herring gulls are capable of object tracking [26] and glaucous-winged gulls (*Larus glaucescens*) have been shown to be quick social learners [37]. This study further extends our understanding of gulls' cognitive capabilities in terms of object and social cues. Our primary finding is that over 90% of pecks were directed toward the item that colour-matched that held by the experimenter. Thus, subjects were able to read human behaviour and make a connection between a stimulus on the ground and that held by the experimenter, allowing gulls to make foraging choices influenced by human behaviour. Learning about a secondary object from the observation of another's behaviour is called stimulus enhancement [27] and requires a different skill set to that demonstrated by observations of object tracking [26].

An increased attention toward human cues in a food-related context is frequently seen in domesticated animals. Both dogs (*Canis lupus familiaris*) and horses (*Equus caballus*) can find hidden food by paying attention to human cues [38–42]. Similarly, while human gaze cues are not sufficient for goats (*Capra hircus*), touching and pointing cues can be used to locate food items [43]. Urban gulls may not be domesticated, but their history of adaptation to anthropogenic environments and frequent interactions with humans may have resulted in a similar increase in attention to and understanding of human food cues.

An alternative, non-exclusive explanation is that kleptoparasitism is a driver for cross-species reading of behaviour. Wild brown skuas (*Catharacta antarctica* ssp. *lonnbergi*), another kleptoparasitic seabird, prefer food that has previously been handled by an experimenter [44] and can follow human behavioural cues [45], which raises questions as to whether frequent contact with humans is a prerequisite for the exploitation of human cues or simply a facilitator, as kleptoparasites may possess a general tendency for paying attention to heterospecific cues.

Whether or not our observed gull behaviour is due to increased contact with anthropogenic scenarios, the pattern of results with age may suggest that lifetime learning plays an important role. Gulls learn to identify valuable food sources from observing conspecifics [21] and juvenile gulls improve foraging skills as they mature [46]. Stimulus enhancement, where observed object-focussed behaviour increases the salience of an object, may explain most of our results. Immature individuals and adults may have paid an equal amount of attention to human food cues, as evidenced by head turns, but adults were more likely to take this interest further by approaching or pecking at the food item. On one hand, this could be explained by a higher pressure on adults to exploit available resources during the breeding season, which partly overlapped with our study period. On the other hand, immature birds may be deterred by competition with adults, in which they are likely to lose; adults chasing away approaching immature individuals was observed on occasion in this study and Monaghan [47] found that the proportion of adult herring gulls foraging at tip sites was significantly higher than the proportion of adults in the population, suggesting that exploitation of human food items may be higher in adults that outcompete immature individuals. Alternatively, immature individuals may lack the skill or boldness to approach a person in possession of food, a notion that is supported by Källander [48] who found that juvenile kleptoparasitic success in black headed gulls (*Larus ridibundus*) increased over time. In this case, if the development of foraging skill depends on social learning, this is another interesting dimension of cognition in gulls [15].

Notably, some individuals were observed moving to the location of the experimenter rather than the presented food items, suggesting that those gulls were motivated by local enhancement [22]. In our case, this would mean that gulls approached because the experimenter eating at that location increased the attractiveness of a point in space, of the experimenter themselves, or of the held food item. This process has been reported in yellow legged gulls (*Larus michahellis*) and Cory's shearwater (*Calonectris borealis*) when identifying suitable foraging areas [49,50], and its presence in gulls has been suggested by Goumas *et al*. [26]. Moreover, some gulls approached the location where the experimenter had been sitting after the trial had ended and the experimenter had moved away.

The distinction between gulls acting under stimulus versus local enhancement may be part of a broader variety in anthropocentric behaviours in urban herring gull populations. Our data agree with existing literature that states that only around a quarter of gulls will interact with anthropogenic food items [8], which is consistent with human–gull interactions requiring skill and boldness and there being a subset of human-foraging specialists within the population. However, those that do interact with people seem to possess a cognitive toolkit that will make the mitigation of human–wildlife conflicts difficult. Our results indicate that observing humans eating a specific food item will not only make that specific item more attractive, but also increase a gull's preference for other items like it. Thus, successful mitigation strategies will have to extend beyond simply preventing the public from feeding gulls but should also take into account their observational skills.

## Data Availability

The data along with a description are available from: https://doi.org/10.25377/sussex.22310716 [51]. The data are provided in electronic supplementary material [52].
